# Patient expectations do matter - experimental evidence on antibiotic prescribing decisions

**DOI:** 10.1093/eurpub/ckac131.188

**Published:** 2022-10-25

**Authors:** S Wang, P Cantarelli, O Groene, N Belle

**Affiliations:** 1 Research and Innovation, OptiMedis, Hamburg, Germany; 2 Management and Healthcare Lab, Sant'Anna, Pisa, Italy; 3 Hamburg Center for Health economics, University of Hamburg, Hamburg, Germany

## Abstract

Inappropriate prescription of antibiotics remains a major contributor to the global antimicrobial resistance crisis despite clear linkages between antibiotic utilization and resistance spread. This study aims to better understand the simultaneous and independent effect of previous prescription behavior, patient expectation, and clinical uncertainty on antibiotic prescribing. This discrete choice experiment was embedded within a routine organizational climate survey administered to all physicians working in the Tuscany healthcare system administered between Nov 11 and Nov 20, 2019 (Qualtrics). Participants were provided with a patient encounter vignette and subsequently asked to in which of two alternatives they were more likely to prescribe antibiotics. The two alternatives varied in levels of clinical uncertainty, patient expectations, and the physician’s past behavior. We fitted a conditional logistic regression model. Respondents included 1,436 hospital-based physicians, of which 52% were female, 78% practiced in a general hospital setting, and 33% were between the ages of 50 and 59. Results show that the odds of prescribing antibiotics decrease when a patient requests it (OR = 0.80, 95%CI [0.72,0.89]) and increase when the physician has prescribed antibiotics to a patient under similar circumstances previously (OR = 1.15, 95%CI [1.03,1.27]). We found no significant effect of clinical uncertainty on the odds of prescribing antibiotics (OR = 0.96, 95%CI [0.87, 1.07]). We show that patient expectation has a significant negative association with antibiotic prescribing among hospital-based physicians. Our findings inform the design of antibiotic stewardship programs in Tuscany and highlight the importance of cultural context in shaping the physician’s disposition when confronted with patient expectations. We suggest shared decision-making to improve prudent prescribing without compromising on patient satisfaction.

**Key messages:**

• Health administrators should address patient expectations when designing hospital antibiotic stewardship programs.

• Physicians’ past prescribing behaviour influences antibiotic prescribing decisions and should be considered during intervention design.

